# Virtual Reality in Clinical Nursing Practice Over the Past 10 Years: Umbrella Review of Meta-Analyses

**DOI:** 10.2196/52022

**Published:** 2023-11-23

**Authors:** Yanjie Hu, Xingzhu Yuan, Peiling Ye, Chengting Chang, Yue Han Hu, Weihua Zhang, Ka Li

**Affiliations:** 1 West China Hospital/West China School of Nursing, Sichuan University, Chengdu, China; 2 School of Computer Science, Sichuan University, Chengdu, China

**Keywords:** virtual reality, clinical nursing, artificial intelligence, AI-assisted medical rehabilitation, health promotion, umbrella review

## Abstract

**Background:**

Virtual reality (VR) has shown promising levels of effectiveness in nursing education, pain management, and rehabilitation. However, meta-analyses have discussed the effects of VR usage in nursing unilaterally and inconsistently, and the evidence base is diffuse and varied.

**Objective:**

We aimed to synthesize the combined evidence from meta-analyses that assessed the effects of nurses using VR technology on nursing education or patient health outcomes.

**Methods:**

We conducted an umbrella review by searching for meta-analyses about VR intervention in clinical nursing practice on Web of Science, Embase, Cochrane, and PubMed, and in reference lists. Eligible studies were published in English between December 1, 2012, and September 20, 2023. Meta-analyses of ≤2 intervention studies and meta-analyses without 95% CI or heterogeneity data were excluded. Characteristic indicators, population information, VR intervention information, and 95% CIs were extracted. A descriptive analysis of research results was conducted to discern relationships between VR interventions and outcomes. *I*^2^ and *P* values were used to evaluate publication bias. AMSTAR (A Measurement Tool to Assess Systematic Reviews) 2 and the GRADE (Grading of Recommendations Assessment, Development, and Evaluation) checklist were used to appraise literature quality.

**Results:**

In total, 768 records were identified; 74 meta-analyses were included for review. The most reported VR study conditions were neuronursing (25/74, 34%), pediatric nursing (13/74, 18%), surgical and wound care (11/74, 15%), oncological nursing (11/74, 15%), and older adult nursing (10/74, 14%). Further, 30% (22/74) of meta-analyses reported publication bias, and 15% (11/74) and 8% (6/74) were rated as “high” based on AMSTAR 2 and the GRADE checklist, respectively. The main outcome indicators among all included meta-analyses were pain (37/214, 17.3%), anxiety (36/214, 16.8%), cognitive function (17/214, 7.9%), balance (16/214, 7.5%), depression (16/214, 7.5%), motor function (12/214, 5.6%), and participation in life (12/214, 5.6%). VR treatment for cognition, pain, anxiety, and depression was effective (all *P* values were *<*.05), while the utility of VR for improving motor function, balance, memory, and attention was controversial. Adverse effects included nausea, vomiting, and dizziness (incidence: range 4.76%-50%). The most common VR platforms were Pico VR glasses, head-mounted displays, the Nintendo Wii, and the Xbox Kinect. VR intervention duration ranged from 2 weeks to 12 months (typically ≥4 wk). VR session length and frequency ranged from 5 to 100 minutes and from 1 to 10 times per week, respectively.

**Conclusions:**

VR in nursing has positive effects—relieving patients’ pain, anxiety, and depression and improving cognitive function—despite the included studies’ limited quality. However, applying VR in nursing to improve patients’ motor function, balance, memory, and attention remains controversial. Nursing researchers need to further explore the effects and standard operation protocols of VR in clinical practice, and more high-quality research on VR in nursing is needed.

## Introduction

Virtual reality (VR) refers to an immersive digital technology that was first conceptualized in the 20th century [1,2] and involves using computer devices and hardware to interact with a specific artificial sensory environment. VR technology can create a standardized, safe, flexible, and virtual environment and provide real-time, strategic, and goal-directed feedback [3]. Because of these advantages, VR technology entered the medical field in 1993 [4]. More and more countries have introduced policies to promote the application of VR technology in the field of health care [5]. Evidence suggests that VR could be beneficial in enhancing the surgical abilities of physicians and minimizing errors during surgical procedures [6]. Additionally, some research indicates that VR may elicit neurophysiological changes, beyond basic distraction, that contribute to its efficacy for pain management [7,8]. The potential of VR indicates that there will be few areas of medicine that do not take advantage of this improved computer interface.

As popular equipment for health care assistance, VR technology plays an important role in the field of clinical nursing, and VR environments are ideally suited to the measurement of many variables of interest in clinical nursing practice. VR was first used by nurses in the field of clinical rehabilitation nursing and gradually became extensively used in the fields of neurological disease, cancer, and wound care [9-11]. Several studies indicate that VR rehabilitation training is more effective among patients with Parkinson disease, especially in improving gait and balance ability [12,13]. VR can also be used as a distraction from pain and anxiety among pediatric patients and patients with cancer. Mohammad and Ahmad [14] found that immersive VR is an effective distraction intervention for managing pain and anxiety among patients with breast cancer. It is also reported that using immersive VR as an auxiliary intervention is more effective than using morphine alone in relieving pain and anxiety [15]. Moreover, the use of VR technology may be an effective, auxiliary, nondrug method for managing kinesiophobia [16].

Although it has been shown in various publications that VR has the potential to help in clinical nursing practice, there remain controversies on the functions, effects, and intervention protocols of VR application in clinical nursing. In addition, the results of multiple meta-analyses that discussed VR intervention effects in different patients are inconsistent [17-19]. Further, the methodological limitations of current evidence impede definitive conclusions regarding the superiority of VR interventions over conventional approaches. A meta-analysis that examined the effect of VR training on patients’ participation noted uncertainty regarding evident publication bias [2], indicating that conclusions regarding the superiority of VR should be made cautiously. Researchers believe that further rigorous research is required to engender robust evidence substantiating the prospective benefits of VR technology [20].

Umbrella reviews can evaluate the strength of the evidence from existing meta-analyses. An umbrella review integrates data and evaluates information on all clinical outcomes, and it can be used to provide a thorough, high-level summary of the evidence landscape for VR application in clinical nursing practice [21]. Given the background presented herein, we performed an umbrella review in which we synthesized and appraised evidence from selected meta-analyses to generate robust conclusions regarding the state of the literature.

## Methods

### Study Design

An umbrella review of meta-analyses was carried out according to the PRISMA (Preferred Reporting Items for Systematic Reviews and Meta-Analyses) reporting guidelines, as described in Checklists 1 and 2. The research questions used to guide this umbrella review were as follows:

1. What is the current scope and extent of VR technology integration in clinical nursing practice?
2. For which clinical nursing issues has VR been principally used as an intervention, and what evidence exists regarding the efficacy and safety of VR in these contexts?
3. What are the primary barriers impeding the broader adoption of VR in clinical nursing settings, and what are the future research directions that may facilitate expanded VR application in nursing practice?

### Inclusion and Exclusion Criteria

The eligibility criteria were as follows: studies that used a meta-analytic method, meta-analyses about VR intervention in clinical nursing practice, and meta-analyses published in English. The exclusion criteria were as follows: meta-analyses that collected ≤2 intervention studies and meta-analyses that had no 95% CI and heterogeneity data.

### Search Strategy

We conducted the umbrella review by searching Web of Science, Embase (Ovid), Cochrane Library, PubMed, and relevant reference lists. Eligible studies were published between December 1, 2012, and September 1, 2023. The searches were rerun on September 20, 2023, to identify any recent publications. We reran the searches before the submission of this paper, and no extra literature was found. We searched for publications that included the following terms (including variations of these terms) in the title, abstract, and keywords list: *virtual reality*, *VR*, *virtual environment*, *immersive*, *nursing*, *care*, *meta-analysis*, and *review*. We also searched the reference lists of the most recent systematic reviews and meta-analyses. The literature retrieval strategy is shown in Multimedia Appendix 1.

### Data Extraction and Collection

One researcher conducted the electronic database searches, eliminated duplicates and titles that were clearly outside the scope of the umbrella review, and then uploaded the remaining citations to NoteExpress version 3.7.09258 (Aegean Software Corp). Two reviewers independently examined the remaining full-text articles to identify those that met the inclusion and exclusion criteria. If there were multiple meta-analyses with the same research objectives and outcome indicators, the one with the highest quality score was selected. Any disagreements were resolved through discussion with a third reviewer.

Data were extracted and managed independently by 2 reviewers using a predefined extraction form. Any concerns were discussed with a third reviewer.

The following data were extracted: (1) characteristic indicators of meta-analyses (first author, year of publication, study design, study period, and number of component primary studies); (2) characteristics of primary studies (trial design, number of participants, and sectionalization); (3) population information (diagnosis and sample size); (4) VR intervention information (VR platform, population, and intervention course); and (5) statistical summaries (outcomes and effect measures with 95% CIs and heterogeneity).

### Data Analysis

We did not reanalyze the other data or primary studies included in the meta-analyses because of the clinical and statistical heterogeneity between the study objectives and outcome indicators of the meta-analyses, and many of the articles did not provide the original data of the original clinical studies. As a result, a descriptive analysis was conducted to encapsulate the impact of VR on clinical nursing practice over the past decade. The process of executing this descriptive analysis involved presenting research results, such as participant details, outcomes, sample sizes, and study designs. These data were meticulously extracted into a predefined Excel (Microsoft Corp) form by YH and XY. Following this, the two authors conducted a thorough review and verification of the collected data to ensure their accuracy and reliability. In instances of disagreement, a consensus was achieved through discussion. In particular, the theme—discerning the relationships between VR interventions and patient outcomes—was summarized.

### Quality Evaluation of Included Literature

AMSTAR (A Measurement Tool to Assess Systematic Reviews) 2 [22] and the GRADE (Grading of Recommendations Assessment, Development, and Evaluation) checklist [23] were used to independently evaluate the methodological quality of the selected meta-analyses. Two researchers separately evaluated the evidence strength of meta-analyses. If there was disagreement, another researcher was asked to make a judgment.

We reviewed the full texts and supplementary materials of included meta-analyses. Two researchers extracted the estimated pooled effect and heterogeneity of each outcome reported in the meta-analyses. The estimated pooled effect, along with its 95% CI, for each included meta-analysis was extracted. We used the *I*^2^ metric to assess heterogeneity (<25%: might show no heterogeneity; 25%-50%: might show moderate heterogeneity; 50%-75%: might show substantial heterogeneity; 75%-100%: considerable heterogeneity), and heterogeneity and *P* values (significant at *P<*.05) were used to assess publication bias.

### Ethical Considerations

The protocol for this umbrella review was originally registered with PROSPERO in December 2022 (registration number: CRD42022381382). No ethical approval was needed, as we used data from published studies.

## Results

### Study Selection

**Figure 1.**
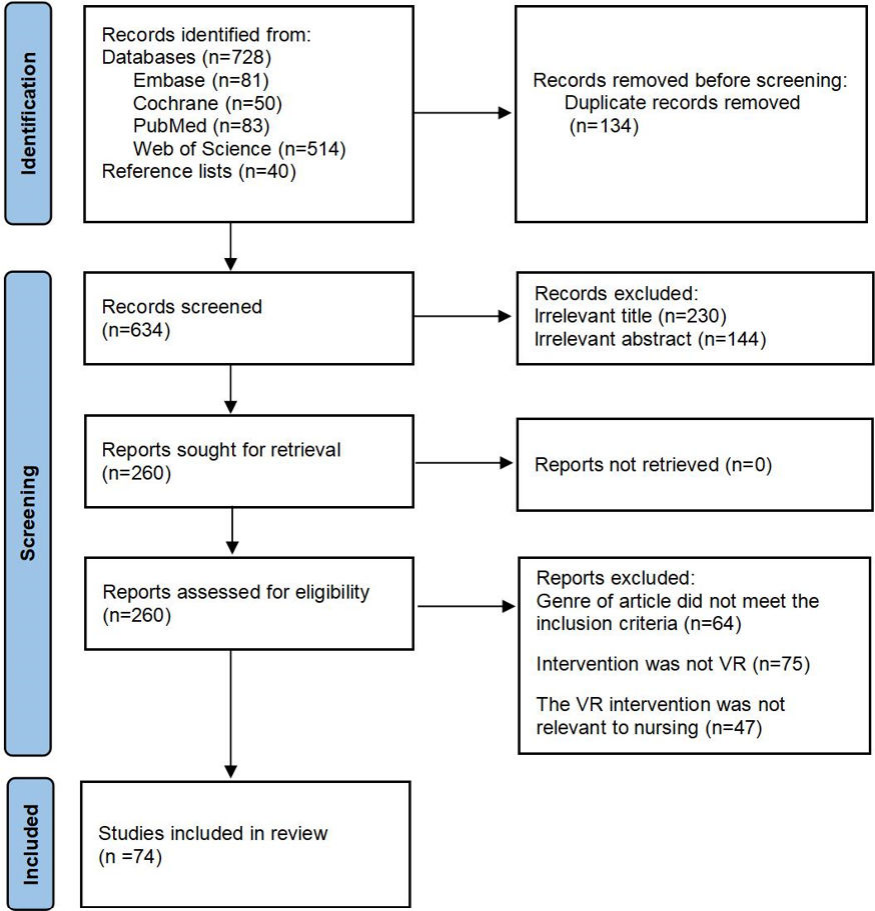
PRISMA (Preferred Reporting Items for Systematic Reviews and Meta-Analyses) flow diagram for the search and selection of the eligible studies included in the umbrella review. Studies were identified via databases and registers. VR: virtual reality.

The electronic literature search identified 768 records from 4 databases and the reference lists of included reviews. We screened the titles and abstracts of 634 records after removing 134 duplicate records. A total of 260 reviews remained after the titles and abstracts were screened for inclusion against predefined criteria. After reading the full texts, 74 articles were finally selected. Further details can be found in Figure 1.

### Basic Features of Included Studies

The included meta-analyses were meta-analyses of randomized controlled trials (RCTs), cross-over studies, pre-post studies, interrupted time series studies, quasi-controlled trials, case reports, controlled trials, or controlled clinical trials, and details of all included meta-analyses are listed in Tables1  2.

**Table 1.** Characteristics of all included meta-analyses.

Study (author, year)	Period	Study design analyzed	Population analyzed	Sample size, n (VR^a^ group and control group)
Chen et al [24], 2014	1993-2013	RCT^b^/CR^c^	Neurology	128 (100 and 28)
Välimäki et al [25], 2014	1994-2012	RCT	Pediatrics	156 (80 and 76)
Cheok et al [26], 2015	2010-2014	RCT	Neurology	166 (84 and 84)
Chen et al [27], 2015	2008-2015	RCT	Neurology	76 (38 and 38)
Christensen et al [28], 2016	1997-2013	RCT	Older adults	343 (183 and 160)
Howes et al [29], 2017	2003-2015	RCT	Older adults	719 (364 and 355)
Bukola and Paula [30], 2017	2002-2013	RCT	Cancer	144 (71 and 73)
Laver et al [31], 2017	2004-2017	RCT	Neurology	2470 (N/A^d^)
Scheffler et al [32], 2018	1981-2013	RCT	Sugery/wound	178 (135 and 43)
Wang et al [33], 2019	2003-2018	RCT	Osteoarthritis	126 (63 and 63)
Eijlers et al [18], 2019	1999-2018	RCT/ITSS^e^	Pediatrics	N/A
Lei et al [13], 2019	2011-2018	RCT	Neurology	555 (N/A)
Kim et al [34], 2019	2003-2017	RCT/CT^f^	Neurology	271 (150 and 121)
Perrochon et al [35], 2019	2013-2017	RCT	Neurology	613 (306 and 305)
Zeng et al [36], 2019	2013-2018	RCT/CCT^g^/PPS^h^	Cancer	225 (137 and 136)
Corregidor-Sanchez et al [37], 2020	2011-2019	RCT	Older adults	491 (336 and 155)
Custodio et al [38], 2020	2012-2018	RCT	Pediatrics	N/A
De Miguel-Rubio et al [19], 2020	2010-2018	RCT/COS^i^/CR	Pediatrics	188 (131 and 57)
De Miguel-Rubio et al [39], 2020	2012-2018	RCT	Pediatrics	150 (81 and 69)
De Miguel-Rubio et al [40], 2020	2011-2018	CT	Pediatrics	103 (57 and 46)
Ding et al [41], 2020	2013-2019	RCT	Surgery/wound	723 (363 and 360)
Dominguez-Tellez et al [42], 2020	2007-2018	RCT	Neurology	874 (440 and 434)
Georgescu et al [43], 2020	2000-2018	RCT	Surgery/wound	1452 (659 and 793)
Lauwens et al [44], 2020	2000-2019	RCT/CR	Surgery/wound	142 (N/A)
Lopez-Valverde et al [45], 2020	2001-2009	RCT	Pediatrics	891 (485 and 406)
Low et al [46], 2021	2004-2019	RCT	Pediatrics	297 (154 and 143)
Czech et al [47], 2021	2002-2018	RCT	Pediatrics	617 (394 and 223)
Fandim et al [48], 2021	2003-2019	RCT	Neurology	1233 (629 and 604)
Chen et al [49], 2021	2007-2020	RCT	Neurology	1428 (656 and 772)
Jung et al [50], 2021	2010-2021	RCT	Neurology	41 (21 and 21)
Li et al [51], 2021	2012-2020	RCT	Neurology	836 (426 and 410)
Sajeev et al [52], 2021	2005-2020	RCT	Pediatrics	1085 (537 and 548)
Yen and Chiu [53], 2021	2012-2020	RCT	Older adults	1022 (503 and 519)
Zhang et al [54], 2021	2011-2019	RCT	Neurology	3540 (1783 and 1757)
Zhang et al [55], 2021	2011-2020	RCT	Neurology	894 (414 and 480)
Zhong et al [56], 2021	2014-2021	RCT	Neurology	744 (374 and 370)
Blasco-Peris et al [57], 2022	2006-2021	RCT	Older adults	152 (81 and 71)
Bu et al [58], 2022	2013-2021	RCT/QCT^j^/PPS	Cancer	478 (330 and 148)
Chan et al [59], 2022	2012-2021	RCT	Neurology	48 (16 and 32)
Chen et al [60], 2022	2007-2021	RCT	Neurology	789 (380 and 409)
Chen et al [61], 2022	2016-2021	RCT	Neurology	1149 (571 and 578)
Chen et al [62], 2023	2017-2019	RCT	Surgery/wound	529 (263 and 266)
Czech et al [2], 2022	2005-2021	RCT/COS	Surgery/wound	587 (481 and 106)
Huber et al [63], 2022	2008-2020	RCT	Neurology	214 (108 and 106)
Kim et al [64], 2022	2012-2021	RCT	Neurology	793 (357 and 436)
Mo et al [65], 2022	2012-2021	RCT/PPS	Cancer	N/A
Obrero-Gaitán et al [66], 2022	2015-2022	RCT/PPS	Cancer	1547 (783 and 764)
Saliba et al [67], 2022	2006-2020	RCT	Pediatrics	930 (468 and 462)
Simonetti et al [68], 2022	2017-2019	RCT	Surgery/wound	602 (297 and 305)
Suleiman-Martos et al [69], 2022	2012-2021	RCT	Surgery/wound	603 (300 and 303)
Tas et al [20], 2022	1999-2020	RCT/ITSS	Pediatrics	1695 (N/A)
Thi et al [70], 2022	1998-2017	RCT	Cancer	201 (92 and 109)
Wang et al [16], 2023	2015-2021	RCT	Kinesiophobia	488 (208 and 280)
Wang et al [71], 2022	2019-2021	RCT	Surgery/wound	1146 (571 and 575)
He et al [72], 2022	2007-2021	RCT	Surgery/wound	1258 (588 and 670)
Zhang et al [73], 2022	2016-2020	RCT/QCT	Cancer	443 (236 and 207)
Zhang et al [74], 2022	2016-2019	RCT	Neurology	609 (303 and 306)
Wong et al [75], 2023	2002-2022	RCT	Anxiety	720 (N/A)
Liu et al [76], 2023	2017-2019	RCT	Neurology	752 (N/A)
Hao et al [77], 2023	2017-2022	RCT/CCT	Cancer	799 (N/A)
Kodvavi et al [78], 2023	2018-2022	RCT	Surgery/wound	375 (188 and 187)
Parra et al [79], 2023	2011-2022	RCT	Neurology	898 (N/A)
Chen et al [80], 2023	2008-2022	RCT	Older adults	482 (N/A)
Kavradim et al [81], 2023	2013-2022	RCT	Older adults	739 (369 and 370)
Shen et al [82], 2023	2012-2021	RCT	Neurology	423 (N/A)
Bok et al [83], 2023	2009-2017	RCT	Neurology	761 (383 and 378)
Tian et al [84], 2023	2004-2021	RCT/QCT	Cancer	797 (N/A)
Yan et al [85], 2023	2012-2022	RCT	Pediatrics	818 (404 and 414)
Gao et al [86], 2023	2017-2022	RCT	Surgery/wound	892 (N/A)
Wu et al [87], 2023	2018-2022	RCT	Cancer	425 (202 and 223)
Ren et al [88], 2023	2011-2021	RCT	Older adults	2404 (1181 and 1223)
Lee et al [89], 2023	Not reported	RCT	Older adults	1095 (N/A)
Burrai et al [90], 2023	2003-2022	RCT/COS	Cancer	459 (222 and 237)
Percy et al [91], 2023	2015-2021	RCT	Older adults	265 (162 and 103)

a VR: virtual reality.

b RCT: randomized controlled trial.

c CR: case report.

d N/A: not applicable.

e ITSS: interrupted time series study.

f CT: controlled trial.

g CCT: controlled clinical trial.

h PPS: pre-post study.

i COS: cross-over study.

j QCT: quasi-controlled trial.

**Table 2.** Outcomes of included meta-analyses.

Studies (author, year) and outcomes		Estimated effect (95% CI)	Heterogeneity (*I*^2^), %	Publication bias, *P* value
**Chen et al [** **24** **], 2014**				
	Motion	1.00 (0.45 to 1.56)	56	N/A^a^
**Välimäki et al [** **25** **], 2014**				
	Cognition	4.67 (–1.76 to 11.1)	8	N/A
	Satisfaction	5.1 (1.03 to 9.17)	0	N/A
**Cheok et al [** **26** **], 2015**				
	Balance	0.39 (–0.25 to 1.04)	85	.02
**Chen et al [** **27** **], 2015**				
	BI^b^	−0.5 (−2.4 to 0.13)	27	N/A
	Balance	−0.5 (−0.7 to 0.37)	0	N/A
**Christensen et al [** **28** **], 2016**				
	HbA_1c_^c^	–0.10 (–0.33 to 0.14)	0	.71
**Howes et al [** **29** **], 2017**				
	Balance	0.56 (0.25 to 0.87)^d^	66	.10
	Mobility	–0.12 (–0.48 to 0.03)	6	.54
	Cognition	–0.65 (–1.03 to –0.28)	58	.07
	Fear	0.28 (–0.50 to 1.05)	0	.55
**Bukola and Paula [** **30** **], 2017**				
	Pain	–0.64 (–1.10 to –0.17)^d^	61.3	N/A
**Laver et al [** **31** **], 2017**				
	Gait	0.09 (–0.04 to 0.22)^d^	10	N/A
	Balance	0.39 (–0.09 to 0.86)^d^	10	N/A
	Mobility	–4.76 (–8.91 to –0.61)^d^	50	N/A
	Motion	0.01 (–0.60 to 0.61)^d^	0	N/A
**Scheffler et al [** **32** **], 2018**				
	Pain	0.69 (0.40 to 0.98)^d^	72	No
	Anxiety	0.36 (0.20 to 0.52)^d^	0	No
**Wang et al [** **33** **], 2019**				
	Pain	–0.25 (–0.48 to –0.02)^d^	32	N/A
	Balance	29.36 (–6.99 to 65.71)^d^	88	N/A
**Eijlers et al [** **18** **], 2019**				
	Pain	1.30 (0.68 to 1.91)	93	No
	Anxiety	1.32 (0.21 to 2.44)	96	No
**Lei et al [** **13** **], 2019**				
	Gait	0.15 (–0.50 to 0.19)	32	High
	Balance	0.22 (0.01 to 0.42)^d^	0	High
	Mobility	–1.95 (–2.81 to –1.06)^d^	60	High
**Kim et al [** **34** **], 2019**				
	Cognition	0.42 (0.24 to 0.60)	5	.001
	Physical fitness	0.41 (0.16 to 0.65)	0	<.001
	Emotion	0.14 (0.07 to 0.36)	36	.20
	Execution	0.07 (0.34 to 0.49)	66	.34
	Feasibility	0.12 (0.10 to 0.34)	54	.30
**Perrochon et al [** **35** **], 2019**				
	Motion	0.53 (–0.35 to 1.42)	0	.58
	Satisfaction	0.08 (0.03 to 0.13)	0	.001
	Safety	0.17 (–0.02 to 0.36)	1	.15
**Zeng et al [** **36** **], 2019**				
	Anxiety	–3.03 (–6.20 to 0.15)	95	.001
	Depression	–1.11 (–3.17 to 0.96)	0	.51
	Fatigue	–2.50 (–4.28 to –0.73)^d^	16	.27
	Pain	–1.63 (–4.15 to 0.89)	94	.001
	Cognition	0.40 (4.64 to 5.44)	36	N/A
**Corregidor-Sanchez et al [** **37** **], 2020**				
	Motion	–0.56 (–0.90 to –0.21)	55	High
**Custodio et al [** **38** **], 2020**				
	Pain	–0.46 (–0.91 to –0.01)^d^	82	No
	Anxiety	–3.37 (–4.57 to –2.81)^d^	71	No
**De Miguel-Rubio et al [** **19** **], 2020**				
	Balance	3.42 (2.54 to 4.29)^d^	70	N/A
**De Miguel-Rubio et al [** **39** **], 2020**				
	BI	–0.37 (–1.38 to 0.64)	68	N/A
**De Miguel-Rubio et al [** **40** **], 2020**				
	ROM^e^	–0.93 (–1.95 to 0.09)	0	N/A
	Balance	–0.27 (–0.82 to 0.27)	56	N/A
**Ding et al [** **41** **], 2020**				
	Pain	–0.64 (–1.05 to –0.22)	69	N/A
**Dominguez-Tellez et al [** **42** **] , 2020**				
	Motion	1.53 (0.51 to 2.54)^d^	92	No
	QOL^f^	2.37 (–0.25 to 4.98)	0	No
	MBI^g^	0.77 (0.05 to 1.49)^d^	95	No
**Georgescu et al [** **43** **], 2020**				
	Pain	0.95 (0.32 to 1.57)^d^	86	No
	Cognition	0.94 (0.33 to 1.56)^d^	51	No
**Lauwens et al [** **44** **], 2020**				
	Pain	0.94 (0.92 to 1.27)	52	N/A
**Lopez-Valverde et al [** **45** **], 2020**				
	Pain	–0.67 (–1.58 to 0.24)	0	.18
	Anxiety	0.20 (–0.48 to 0.87)	0	.54
**Low et al [** **46** **], 2021**				
	Satisfaction	0.45 (–0.07 to 0.97)	70	N/A
	Dissatisfaction	0.72 (0.25 to 1.20)	44	N/A
**Czech et al [** **47** **], 2021**				
	Pain	−2.85 (−3.57 to −2.14)^d^	0	N/A
	Fear	−0.19 (−0.58 to 0.202)	94	N/A
	Anxiety	N/A	93	N/A
	Satisfaction	N/A	83	N/A
**Fandim et al [** **48** **], 2021**				
	Motion	–0.08 (–0.45 to 0.29)	6	No
	Balance	1.43 (0.61 to 2.24)^d^	53	No
**Chen et al [** **49** **], 2021**				
	BI	0.23 (0.13 to 0.34)^d^	0	.28
**Jung et al [** **50** **], 2021**				
	Cognition	0.45 (0.20 to 0.71)	42	.58
**Li et al [** **51** **], 2021**				
	Balance	0.66 (N/A)	64	.23
	QOL	–0.28 (N/A)	0	<.001
	MBI	0.62 (N/A)	0	<.001
	Depression	–0.75 (N/A)	80	.75
**Sajeev et al [** **52** **], 2021**				
	Pain	–0.43 (–0.67 to –0.20)^d^	81	N/A
	Anxiety	–0.61 (–0.88 to –0.34)^d^	89	N/A
**Yen and Chiu [** **53** **], 2021**				
	Cognition	0.53 (0.32 to 0.73)^d^	2	.57
	Memory	0.51 (0.06 to 0.96)^d^	58	.50
	Attention	0.49 (–0.10 to 1.08)	88	.44
	Execution	0.05 (–0.37 to 0.46)	81	.79
	Depression	–1.00 (–1.51 to –0.45)	55	.90
**Zhang et al [** **54** **], 2021**				
	Motion	3.01 (1.91 to 4.11)	70	N/A
	Balance and gait	3.51 (2.10 to 4.92)	80	N/A
	Cognition	0.81 (−0.41 to 2.03)	66	N/A
	MBI	7.02 (4.96 to 9.08)	18	N/A
**Zhang et al [** **55** **], 2021**				
	Cognition	0.32 (–0.43 to 1.06)	89	.29
	Attention	0.78 (0.23 to 1.33)^d^	6	.04
	Depression	0.20 (–0.25 to 0.64)	16	.55
	QOL	3.01 (1.51 to 4.31)	12	.17
**Zhong et al [** **56** **], 2021**				
	Cognition	0.42 (0.04 to 0.79)	3	No
	Attention	0.09 (−0.26 to 0.44)	0	No
**Blasco-Peris et al [** **57** **], 2022**				
	QOL	0.22 (−0.37 to 0.81)	3	N/A
	Depression	0.17 (−0.36 to 0.70)	0	N/A
**Bu et al [** **58** **], 2022**				
	Anxiety	–6.47 (–7.21 to –5.73)^d^	83	No
	Depression	–4.27 (–4.64 to –3.91)^d^	0	No
	Pain	–1.32 (–2.56 to –0.09)^d^	87	No
	Fatigue	8.80 (8.24 to 9.36)^d^	0	No
**Chan et al [** **59** **], 2022**				
	Balance	0.12 (–0.66 to 0.89)^d^	0	N/A
	Motion	0.13 (–0.65 to 0.91)^d^	0	N/A
**Chen et al [** **60** **], 2022**				
	Anxiety	–0.35 (–0.70 to 0.01)	0	N/A
	Depression	–0.48 (–0.84 to –0.12)^d^	0	N/A
**Chen et al [** **61** **], 2022**				
	Cognition	3.00 (2.28 to 3.71)	7	No
	BI	6.14 (4.56 to 7.72)	0	No
	MBI	6.06 (1.27 to 10.85)	86	No
**Chen et al [** **62** **], 2023**				
	Anxiety	−0.91 (−1.43 to −0.39)^d^	86	Low
**Czech et al [** **2** **], 2022**				
	Pain	–0.47 (–0.78 to –0.15)^d^	41	N/A
	ROM	0.44 (−0.23 to 1.11)	50	N/A
**Huber et al [** **63** **], 2022**				
	Cognition	0.43 (0.22 to 0.64)^d^	24	N/A
**Kim et al [** **64** **], 2022**				
	Depression	−0.54 (−0.79 to –0.29)	73	No
**Mo et al [** **65** **], 2022**				
	Pain	–0.59 (–1.15 to –0.04)	78	No
	Fatigue	–0.53 (–0.88 to –0.18)	15	No
	Depression	–0.60 (–1.04 to –0.15)	29	No
	Satisfaction	–0.68 (–1.25 to –0.11)	55	No
**Obrero-Gaitán et al [** **66** **], 2022**				
	Pain	–1.03 (–1.52 to –0.54)^d^	7	.09
	Anxiety	–1.79 (–2.7 to –0.91)^d^	32	.55
	Depression	–2.7 (–4.39 to –0.99)^d^	46	.92
	QOL	0.76 (0.42 to 1.11)^d^	0	.90
**Saliba et al [** **67** **], 2022**				
	Pain	2.54 (0.14 to 4.93)^d^	99	N/A
	Anxiety	0.89 (0.16 to 1.63)^d^	95	N/A
**Simonetti et al [** **68** **], 2022**				
	Anxiety	–0.34 (–0.62 to –0.11)^d^	39	No
**Suleiman-Martos et al [** **69** **], 2022**				
	Anxiety	–10.62 (–13.85 to –7.39)^d^	84	No
**Tas et al [** **20** **], 2022**				
	Pain	–0.67 (–0.89 to 0.45)^d^	68	.30
	Anxiety	–0.74 (–1.00 to 0.48)^d^	59	.56
**Thi et al [** **70** **], 2022**				
	Pain	–0.93 (–2.63 to 0.76)	86	N/A
**Wang et al [** **16** **], 2023**				
	Fear	−0.53 (−0.90 to −0.17)^d^	64	N/A
**Wang et al [** **71** **], 2022**				
	Fear	−1.52 (−2.18 to –0.86)	51	N/A
	Anxiety	−2.79 (−4.07 to –1.54)	0	N/A
	Pain	−2.17 (−3.37 to –0.97)	92	N/A
**He et al [** **72** **], 2022**				
	Pain	–1.13 (–2.01 to –0.26)^d^	97	No
**Zhang et al [** **73** **], 2022**				
	Anxiety	–2.07 (–3.81 to –0.34)	95	N/A
	Fatigue	–0.92 (–4.47 to 2.62)	99	N/A
**Zhang et al [** **74** **], 2022**				
	BI	0.31 (0.10 to 0.51)	31	No
	Grip	0.40 (0.08 to 0.71)	0	No
	Motion	0.71 (0.43 to 0.99)	0	No
**Wong et al [** **75** **], 2023**				
	Anxiety	–1.17 (–2.06 to –0.28)^d^	42	N/A
**Liu et al [** **76** **], 2023**				
	Depression	–0.75 (–1.35 to –0.15)	92.2	N/A
**Hao et al [** **77** **], 2023**				
	Pain	–1.53 (–2.55 to –0.50)	34	N/A
	Fatigue	0.86 (0.44 to 1.28)	97	N/A
	Anxiety	–3.02 (–5.27 to –0.77)	34	N/A
	Motion	–0.66 (–1.02 to –0.31)	47	N/A
	QOL	0.53 (0.14 to 0.93)	34	N/A
**Kodvavi et al [** **78** **], 2023**				
	Anxiety	–0.73 (–1.08 to –0.39)^d^	22	Low
	Pain	–0.25 (–0.44 to –0.05)^d^	67	Low
**Parra et al [** **79** **], 2023**				
	Loss	0.13 (0.02 to 0.24)	N/A	Low
**Chen et al [** **80** **], 2023**				
	Balance	0.62 (0.29 to 0.95)	68	Low
	Cognition	0.90 (0.61 to 1.19)	16	Low
**Kavradim et al [** **81** **], 2023**				
	Anxiety	–0.85 (–1.55 to –0.14)	91.92	High
	Motion	0.54 (0.01 to 1.08)^d^	1.702	High
	Stress	–0.36 (–0.60 to –0.11)	0	High
	Depression	–0.39 (–0.68 to –0.11)	73.979	High
**Shen et al [** **82** **], 2023**				
	Balance	1.35 (0.58 to 1.86)	44	N/A
	MBI	5.26 (1.70 to 8.82)	72	N/A
**Bok et al [** **83** **], 2023**				
	Balance	–0.113 (–0.547 to 0.32)	73.3	N/A
**Tian et al [** **84** **], 2023**				
	Anxiety	–4.93 (–8.00 to –1.87)	NA	High
	Depression	–2.89 (–5.46 to –0.32)	7.05	High
	Pain	–1.2 (–2.05 to –0.50)	66	High
	Cognition	2.69 (1.95 to 3.44)	10	High
	Motion	–0.79 (–5.00 to 3.42)	25	High
	Grip	0.38 (–0.19 to 0.96)	9	High
**Yan et al [** **85** **], 2023**				
	Anxiety	–1.74 (–2.46 to –1.02)	95	.75
	Pain	–1.5 (–2.22 to –0.91)	91	.81
	Heart rate	–10.54 (–20.26 to –0.81)	99	.60
**Gao et al [** **86** **], 2023**				
	Anxiety	–0.77 (–1.24 to –0.31)	85	N/A
**Wu et al [** **87** **], 2023**				
	Anxiety	–0.83 (–1.25 to –0.42)	82	N/A
	Pain	–0.86 (–1.36 to –0.35)	85	N/A
	Depression	–0.46 (–0.74 to –0.18)	76	N/A
	Fear	–0.82 (–1.60 to –0.03)	69	N/A
	Distress	–1.16 (–1.96 to –0.37)	72	N/A
	QOL	1.01 (–0.67 to 2.70)	94	N/A
**Ren et al [** **88** **], 2023**				
	Motion	1.30 (0.08 to 2.51)	88	.19
**Lee et al [** **89** **], 2023**				
	Balance	0.62 (0.14 to 1.10)	80.92	High
**Burrai et al [** **90** **], 2023**				
	Anxiety	–6.57 (–11.59 to –1.54)	92	N/A
	Fatigue	–0.18 (–0.46 to 0.09)	0	N/A
	Pain	–1.95 (–6.96 to 3.06)	99	N/A
**Percy et al [** **91** **], 2023**				
	Motion	−0.91 (−1.38 to −0.44)	95	N/A
	Balance	−0.54 (−1.80 to 0.71)	74	N/A

a N/A: not applicable.

b BI: Barthel Index.

c HbA_1c_: hemoglobin A_1c_.

d Significant at the *P*<.05 level.

e ROM: range of motion.

f QOL: quality of life.

g MBI: modified Barthel Index.

Among all included meta-analyses, the most common participant groups investigated were neuronursing (25/74, 34%), pediatric nursing (13/74, 18%), surgical and wound care (11/74, 15%), oncological nursing (11/74, 15%), and older adult nursing (10/74, 14%) populations. The number of participants in each meta-analysis ranged from 103 to 3540, and 214 outcome indicators were found. The main outcome indicators in all included meta-analyses were pain (37/214, 17.3%), anxiety (36/214, 16.8%), cognitive function (17/214, 7.9%), balance (16/214, 7.5%), depression (16/214, 7.5%), motor function (12/214, 5.6%), and participation in life (12/214, 5.6%), among others. The distribution of populations and outcome indicators of VR application in nursing is shown in Multimedia Appendix 2.

### Quality of Included Reviews

#### Publication Bias

With regard to publication bias, 30% (22/74) of meta-analyses reported publication bias, 23% (17/74) had no publication bias, and 47% (35/74) did not have such data available. Specific data are shown in Tables1  2.

#### GRADE Classification and AMSTAR 2 Score

The meta-analyses were classified into 4 levels, with approximately 15% (11/74) rated as “high,” approximately 28% (21/74) rated as “moderate,” approximately 39% (29/74) rated as “low,” and approximately 18% (13/74) rated as “very low” via AMSTAR 2. The main reasons for lower ratings were that many meta-analyses neither evaluated publication bias nor drafted research protocols in advance, and only a few meta-analyses reported the sources of funding for the studies included in the analysis. Approximately 49% (36/74) of meta-analyses were rated as “very low,” approximately 34% (25/74) were rated as “low,” approximately 9% (7/74) were rated as “moderate,” and approximately 8% (6/74) were rated as “high” via the GRADE system. The detailed results of the AMSTAR 2 and GRADE evaluations are presented in Multimedia Appendices 3 and 4.

### VR Application in Clinical Nursing Practice

#### VR in Neuronursing

A total of 25 articles focused on the effectiveness of VR in neurological nursing. These studies mainly concentrated on motor function, cognition, depression, participation in life, and quality of life. Of these studies, 18 included RCTs. The results showed that VR intervention for cognitive function [61] and depression [51] was effective (*P*<.05). However, its efficacy for motor function, participation in life, and quality of life was controversial. Chan et al [59] reported that VR training was more effective than traditional rehabilitation training in improving balance function (standardized mean difference [SMD] 0.22, 95% CI 0.01-0.42; *P*=.04) and mobility (mean difference −1.95, 95% CI −2.81 to –1.08; *P*<.01), while a study by Parra et al [79] showed that no statistical differences were found in balance and gait between patients with Parkinson disease in the VR group and those in the control group (odds ratio 0.83, 95% CI 0.62-1.12). The differences between meta-analyses were probably the result of the different heterogeneous populations or the interventions being used at different points in the disease course. Heterogeneity was low for depression and cognitive function (range 0.01-0.24).

#### VR in Pediatric Nursing

A total of 13 meta-analyses focused on children and were mainly about nursing for pain, anxiety, and fear. The results of these meta-analysis studies showed that VR technology intervention by nurses to reduce pain and anxiety was effective for most patients. For example, VR was effective in reducing dental anxiety (SMD −1.74, 95% CI −2.46 to –1.02; *P*<.001; *I*^2^=95%) and pain (SMD −1.57, 95% CI −2.22 to –0.91; *P*<.001; *I*^2^=91%) among pediatric patients in a study by Yan et al [85]. Further, Eijlers et al [18] stated that when VR was applied as a distraction during venous access or during dental, burn, or oncological care in pediatric nursing, pain (SMD 1.30, 95% CI 0.68-1.91) and anxiety (SMD 1.32, 95% CI 0.21-2.44) were reduced. However, the effect of VR on fear was controversial [25,39]. In a meta-analysis by Saliba et al [67], VR seemed to be useful in reducing the fear of 648 children with burns (SMD 0.89, 95% CI 0.16-1.63; *P*=.02), but they found that there was significant heterogeneity among included studies. Czech et al [47] considered fear scores, and their study revealed no significant differences between the VR and no VR conditions. Heterogeneity was moderate or substantial in many studies (range 0.56-0.99), which may be the result of the different assessment scales used in each included study.

#### VR in Surgical and Wound Care

A total of 11 meta-analyses focused on surgical and wound care. The results of these meta-analysis studies showed that VR technology intervention for pain [43] (SMD 0.95, 95% CI 0.32-1.57) and anxiety [32] (SMD 0.36, 95% CI 0.20-0.52) was effective. Heterogeneity was low for anxiety (range 0.00-0.23) [2] but substantial for pain (range 0.44-0.80) [72]. Georgescu et al [43] summarized RCTs and found that the cognition of 1452 patients who underwent surgery or wound care statistically improved (SMD 0.94, 95% CI 0.33-1.56; *I*^2^=51%), while the range of motion of patients with burns did not show better progress [2].

#### VR in Oncological Nursing

A total of 11 meta-analyses focused on cancer nursing, including symptom management and rehabilitation nursing, especially for breast cancer. The results of these meta-analysis studies showed that VR technology intervention was effective for pain (mean difference −1.27, 95% CI −2.05 to –0.50; *P*=.001), depression (SMD −2.89, 95% CI −5.46 to –0.32; *P*=.03), and anxiety (SMD −4.93, 95% CI −8.00 to –1.87; *P*=.002) among most patients with breast cancer [66]. However, when combined with its effects for other types of cancer, the effect of VR on pain, anxiety, and depression became controversial. Heterogeneity was substantial for anxiety and pain [73] but low for depression.

#### VR in Gerontological Nursing

A total of 10 meta-analyses focused on gerontological nursing. The results of these meta-analysis studies showed that VR technology intervention by nurses to improve balance (SMD 0.56, 95% CI 0.25-0.87; *P*<.001) was effective [29], while its effectiveness for motion capacity was controversial. Studies showed that virtual motor training had no significant positive effects [37,80]. Heterogeneity was moderate or substantial for exercise capacity, cognitive function, and balance [29,37,80].

### Safety Concerns Regarding VR-­Related Adverse Effects

Only a few papers reported adverse effects caused by using VR. The prevalence of adverse effects in these studies ranged from 4.76% to 50%, and these adverse effects included nausea, vomiting, sickness, dizziness, fatigue, pain, and the risk of losing balance.

### VR Platforms and Intervention Times Among Different Populations

There were various platforms used for VR. We summarize the typical VR technology platforms used for different populations and outcome indicators in Table 3. The most commonly used VR platforms were Pico VR glasses, head-mounted displays, the Nintendo Wii, and the Xbox Kinect. The duration of VR intervention ranged from 2 weeks to 12 months (the most commonly reported durations were ≥4 weeks), and the length and frequency of VR sessions ranged from 5 to 100 minutes and from 1 to 10 times per week, respectively.

**Table 3.** Typical virtual reality (VR) platforms and intervention times among different patients.

Populations and outcome indicators		Platforms	Intervention duration	Session length and frequency
**Neurology**				
	Depression	Nintendo Wii, Xbox Kinect, and HMD^a^	6-12 wk	30-60 min, 2-5 times/wk
	Cognition	Pico UI 4.0, Nintendo Wii, Xbox 360 Kinect, BioMaster 2012, Lokomat, and Oculus	2-12 wk	20-100 min, 2-10 times/wk
	Daily living	Pico UI 4.0, Nintendo Wii, Xbox 360 Kinect, Armeo Spring, RehabMaster, BioMaster 2012, and Toyra VR	2-12 wk	12-60 min, 2-5 times/wk
	QOL^b^	Nintendo Wii, Xbox 360 Kinect, and Armeo Spring	3-8 wk	30-60 min, 2-5 times/wk
	Motion	Pico UI 4.0, Xbox 360 Kinect, Nintendo Wii, HMD, BioFlex, BioMaster, BioRescue, and ReJoyce	2-12 wk	20-90 min, 2-10 times/wk
**Pediatrics**				
	Motion	Pico UI 4.0, Nintendo Wii, and Xbox 360 Kinect	3-12 wk	30-100 min, 2-7 times/wk
	Distraction	Xbox 360 Kinect, HMD, ReJoyce, and Oculus	N/A^c^	5-35 min
**Surgical and wound care**				
	Distraction	Pico UI 4.0 and HMD	4 wk	30 min, 5 times/wk
**Cancer**				
	Anxiety, depression, and pain	Pico UI 4.0 and HMD	N/A	15-20 min
	Motion	HMD, Xbox 360 Kinect, and Nintendo Wii	4-8 wk	20-50 min, 2-5 times/wk
**Older adults**				
	Exercise capacity	HMD, Nintendo Wii, and Xbox 360 Kinect	2 wk to 12 mo	18-45 min, 1-5 times/wk
	Cognition	Xbox 360 Kinect, Nintendo Wii, and BioRescue	8-13 wk	18-30 min, 2-3 times/wk

a HMD: head-mounted display.

b QOL: quality of life.

c N/A: not applicable.

## Discussion

### Principal Findings

Clinical applications of VR in nursing practice predominately concentrate on neurology, pediatrics, oncology, surgical and wound care, and gerontology. Extant evidence indicates that VR interventions confer benefits for alleviating patient anxiety, pain, and depression, especially in pediatrics, among patients with breast cancer, and in wound care processes. VR is also a promising intervention for enhancing cognitive function in neurology patients. However, research on the utility of VR for improving motor function, balance, memory, and attention remains equivocal.

Many meta-analyses suggested that VR in nursing is useful for relieving anxiety and pain in patients, especially in pediatrics, among patients with breast cancer, and in wound care processes. Chen et al [62] found that a VR operation before surgery helped people familiarize themselves with the environment and understand the preoperative preparation procedures, thereby effectively reducing anxiety and improving compliance. Additionally, VR is a promising intervention for procedural pain [18,30], as reported in a study by Addab et al [92]. As a nondrug distraction intervention in nursing, VR can reduce the side effects of pain, depression, and anxiety drugs. However, heterogeneity was substantial in oncology studies and surgical and wound care studies. More high-quality studies are needed to help define the effectiveness of VR in these fields [93]. Further, VR can potentially be used to interfere with cognitive function, as it has been shown to affect cognitive plasticity and neuroplasticity. One study demonstrated plasticity in patients diagnosed with mild cognitive impairment and confirmed that repeated VR situational interactive training improved the excitability of the remaining nerve cells, promoted the functional reorganization of the damaged brain area, and resulted in the formation of new neural circuits, thereby improving the patients’ cognition [94]. In a systematic review and meta-analysis that included 21 original studies (1149 participants), Chen et al [61] suggested that compared with a controlled group, VR training increased cognition because VR training increased the 1-naphthylacetic acid to creatine ratio in the hippocampus, which indicated that VR could improve the levels of hippocampal metabolites in patients with poststroke cognitive impairment, thereby promoting neuronal repair and improving cognitive function. However, several studies indicated that the effects of VR in nursing on cognition need to be clarified in more well-designed RCTs with large sample sizes because of small effect sizes, insufficient outcome indicators, and the low quality of current evidence [34,50,56].

The meta-analysis results showed that VR in nursing probably increased the exercise capacity of individuals undergoing neurologic rehabilitation when compared with usual nursing [60]. In contrast, many meta-analyses showed that there was little confidence that VR in nursing improves physical outcomes in older adults when compared with usual nursing [29,57]. The lack of allocation concealment and the absence of assessor blinding were the main causes of bias. As such, the final grade of the evidence was low, and the conclusions were hard to accept [37]. Some researchers thought that the results of VR therapy showed a trend of gait and upper limb function improvement in individuals [95,96], but Laver et al [31] believed that VR video gaming was not more beneficial than conventional nursing approaches in improving upper limb function, as did Voinescu et al [5]. Further, heterogeneity in protocols, VR task performance, and the different characteristics of the participants could have influenced the results [39]. Zhang et al [54] and Lei et al [13] pointed out that VR training helped to improve balance when compared with usual care. Similarly, Gates et al [97] and Saragih et al [98] concluded that VR training helped to improve balance when compared with usual nursing. All of these included studies did not analyze which subtypes of patients can benefit more from VR training in terms of improved balance, did not assess the long-term effects of VR on balance and mobility, and often included a relatively small number of patients and inadequate control groups [99]. The results need to be interpreted with caution due to the high heterogeneity, small sample sizes, and different outcome measures. In terms of improving memory, even after excluding studies with high heterogeneity, several meta-analyses still concluded that the effects of VR nursing measures on patients’ instantaneous memory, long-term memory, and attention were not statistically significant [31,54,56]. The possible reasons for this are that VR training duration was too short to result in significant improvements [56] and only 6 studies (within the meta-analyses included in this umbrella review) with small samples were used to evaluate the effects of VR on attention.

It is also important to classify and summarize the VR platforms and frequencies of VR use that are commonly used in different nursing scenarios because of their influence on nursing outcomes. Meta-regression analyses showed that the number of VR sessions and the frequency of VR training had statistically significant impacts on balance scores [51]. There are few studies that explain why VR devices and scenes are chosen in a particular context, and there is no comparative analysis of the effects of using different VR devices and scenes in the same session. With regard to these biases, the included studies claimed that heterogeneity was due to the use of VR hardware with multiple types of VR software and variation in the duration of VR therapy sessions [93], which resulted in patients experiencing different levels of immersion, presence, and interactivity. Future research on VR in nursing should focus on providing a detailed report on VR device and platform instructions, the length and frequency of VR sessions, and the duration of VR intervention.

### Comparison to Prior Work

Previous reviews on VR in nursing mostly discussed the impact of VR on nursing education and training at different levels [100,101]. However, the role of VR in clinical nursing practice is also very important. Therefore, we conducted this umbrella review, which aimed to effectively synthesize the combined evidence from meta-analyses that assessed the effects of nurses using VR technology on patients’ health outcomes.

### Strengths

This review followed the PRISMA reporting guidelines; thus, it can be viewed as a transparent and reproducible review. The findings in our review are more likely to be comprehensive and provide evidence for further VR application in clinical nursing practice. This study has a low risk of publication bias because we searched all relevant databases by using a reliable search strategy to identify as many eligible studies as possible. Moreover, we controlled the risk of selection bias by having 2 independent authors perform the research process, including the study selection, data extraction, and quality assessment processes.

### Limitations

First, the quality of many included meta-analyses was not high. Therefore, the conclusions should be considered with care. Second, our study only reports on meta-analyses published in English, which may have resulted in language bias. Third, a quantitative data synthesis was not conducted in this review, given the significant clinical heterogeneity of the included studies in terms of study designs, intervention and population characteristics, and outcome measurements. This was one of the main reasons for the inconclusive research evidence found in this review. Follow-up studies can search for more literature, without strict language restrictions, and try to conduct high-quality quantitative syntheses to establish more reliable conclusions.

### Future Directions

VR games have distinct clinical advantages, as they offer challenging and interesting environments [54]. Authoritative conclusions on the applications of VR in nursing might promote the application of VR games. It is suggested that nursing researchers should improve the quality of research; carry out large-scale, multicenter randomized controlled studies; and make authoritative conclusions on VR in nursing. In addition, the research outcomes of VR application in nursing are mostly subjective indicators, such as anxiety, depression, and satisfaction, and more objective outcome indicators, such as serological indicators, should be mentioned in the future. In the existing research, VR platforms, the duration of VR intervention, and the length and frequency of VR sessions are different. We hope that further research considers establishing standard operation protocols for VR intervention in specific populations.

### Conclusions

Our study comprehensively summarized and indicated the potentially beneficial role of VR intervention in enhancing the management of pain, depression, anxiety, and cognition in neurology, pediatrics, oncology, surgical and wound care, and gerontology; the effects of VR intervention on improving motor function, balance, memory, and attention remain equivocal. However, these findings should be interpreted with caution due to the unsatisfactory quality of the included studies. It is recommended that more research of rigorous methodological quality is necessary to further determine the role of VR in promoting health care outcomes.

## Supplementary material

10.2196/52022 Checklist 1 PRISMA (Preferred Reporting Items for Systematic Reviews and Meta-Analyses) checklist.

10.2196/52022 Checklist 2 PRISMA (Preferred Reporting Items for Systematic Reviews and Meta-Analyses) abstract checklist.

10.2196/52022 Multimedia Appendix 1 Literature retrieval strategy.

10.2196/52022 Multimedia Appendix 2 Distribution of populations and outcome indicators of virtual reality application.

10.2196/52022 Multimedia Appendix 3 Detailed results of the AMSTAR (A Measurement Tool to Assess Systematic Reviews) 2 evaluation.

10.2196/52022 Multimedia Appendix 4 Detailed results of the GRADE (Grading of Recommendations Assessment, Development, and Evaluation) evaluation.

## Abbreviations

AMSTAR: A Measurement Tool to Assess Systematic Reviews
GRADE: Grading of Recommendations Assessment, Development, and Evaluation
PRISMA: Preferred Reporting Items for Systematic Reviews and Meta-Analyses
RCT: randomized controlled trial
SMD: standardized mean difference
VR: virtual reality
