# Supplementary material for: Virtual Reality in Clinical Nursing Practice Over the Past 10 Years: Umbrella Review of Meta-Analyses
Source: JMIR Serious Games. 2023 Nov 23;11:e52022. doi: 10.2196/52022 (PMC10690102; doi:10.2196/52022)
Supplement: Multimedia Appendix 1 [file games-v11-e52022-s003.docx]

**Detailed Retrieval Strategies**

**Embase**

Embase <2012 to 2023 September 20>

1 exp virtual reality/ or virtual reality.mp. 33376

2 exp nursing diagnosis/ or exp international nursing/ or exp newborn nursing/ or exp nursing assistant/ or exp radiology nursing/ or exp perioperative nursing/ or exp nursing information system/ or exp postanesthesia nursing/ or exp nursing care plan/ or exp gastroenterology nursing/ or exp psychiatric nursing/ or exp pediatric intensive care nursing/ or exp geriatric nursing/ or exp rehabilitation nursing/ or exp holistic nursing/ or exp nursing outcome/ or exp nursing expertise/ or exp burn nursing/ or exp nursing evaluation research/ or exp clinical nursing research/ or exp perianesthesia nursing/ or exp rural health nursing/ or exp nursing home/ or exp nursing intervention/ or exp nursing interventions classification/ or exp differentiated nursing practice/ or exp genetics nursing/ or exp primary nursing/ or exp nursing career/ or exp medical surgical nursing/ or exp anesthesia nursing/ or exp Nursing Delirium Screening Scale/ or exp humanistic nursing/ or exp military nursing/ or exp newborn intensive care nursing/ or nursing.mp. or exp nephrology nursing/ or exp nursing practice/ or exp family nursing/ or exp practical nursing/ or exp pediatric nursing/ or exp respiratory nursing/ or exp nursing home personnel/ or exp neuroscience nursing/ or exp cardiovascular nursing/ or exp palliative nursing/ or exp team nursing/ or exp emergency nursing/ or exp nursing informatics/ or exp addictions nursing/ or exp community health nursing/ or exp nursing theory/ or exp nursing home patient/ or exp pediatric oncology nursing/ or exp nursing assessment/ or exp nursing outcomes classification/ or exp nursing process/ or exp intensive care nursing/ or exp community psychiatric nursing/ or exp oncology nursing/ or exp nursing care/ or exp perinatal nursing/ or exp nursing/ or exp nursing organization/ or exp occupational health nursing/ or exp advanced practice nursing/ or exp nursing administration research/ 649460

3 exp meta analysis/ 292563

4 "review"/ or exp "systematic review"/ or exp droperidol/ 3169655

5 3 or 4 3275296

6 1 and 2 and 5 81

**Cochrane**

Search Name: VR colonrectal clinical outcomes

Date Run: 20/09/2023 14:26:15

Comment:

ID Search Hits

#1 (virtual):ti,ab,kw OR (Immersive):ti,ab,kw OR (immersed):ti,ab,kw OR (Kinect):ti,ab,kw (Word variations have been searched) 17093

#2 (nurs*):ti,ab,kw OR (care):ti,ab,kw (Word variations have been searched) 321132

#3 #1 AND #2 4013

#4 limited to Cochrane Reviews 50

**Pubmed**

| S | Query | Search Details | Results |
| --- | --- | --- | --- |
| 8 | ((nurs*[Title/Abstract]) OR (care[Title/Abstract])) AND ("Virtual Reality"[Mesh] OR "Virtual Reality Exposure Therapy"[Mesh] OR "Exergaming"[Mesh]) | (("nurs*"[Title/Abstract] OR "care"[Title/Abstract]) AND ("Virtual Reality"[MeSH Terms] OR "Virtual Reality Exposure Therapy"[MeSH Terms] OR "Exergaming"[MeSH Terms])) AND (meta-analysis[Filter] OR systematicreview[Filter]) | 83 |
| 5 | ((nurs*[Title/Abstract]) OR (care[Title/Abstract])) AND ("Virtual Reality"[Mesh] OR "Virtual Reality Exposure Therapy"[Mesh] OR "Exergaming"[Mesh]) | ("nurs*"[Title/Abstract] OR "care"[Title/Abstract]) AND ("Virtual Reality"[MeSH Terms] OR "Virtual Reality Exposure Therapy"[MeSH Terms] OR "Exergaming"[MeSH Terms]) | 810 |
| 4 | (nurs*[Title/Abstract]) OR (care[Title/Abstract]) | "nurs*"[Title/Abstract] OR "care"[Title/Abstract] | 2,176,987 |
| 1 | "Virtual Reality"[Mesh] OR "Virtual Reality Exposure Therapy"[Mesh] OR "Exergaming"[Mesh] | "Virtual Reality"[MeSH Terms] OR "Virtual Reality Exposure Therapy"[MeSH Terms] OR "Exergaming"[MeSH Terms] | 6,693 |

**Web** **of science**

| Type | Search Query | Database | Results | Date Run |
| --- | --- | --- | --- | --- |
| #1 | meta-analys* (Topic) OR systematic review* (Topic) | Web of Science Core Collection | 577059 | Tue Sep 20 2023 22:33:35 |
| #2 | Virtual Reality (Topic) | Web of Science Core Collection | 75353 | Tue Sep 20 2023 22:33:35 |
| #3 | nurs* (All Fields) OR care (All Fields) | Web of Science Core Collection | 3678104 | Tue Sep 20 2023 22:33:35 |
| #4 | #1 AND #2 AND #3 | Web of Science Core Collection | 527 | Tue Sep 20 2023 22:33:35 |
|  | #1 AND #2 AND #3 and 2023 or 2022 or 2021 or 2020 or 2019 or 2018 or 2017 or 2016 or 2015 or 2014 or 2013 or 2012 (Publication Years) | Web of Science Core Collection | 514 | Tue Sep 20 2023 23:33:35 |
|  |  |  |  |  |
