# Supplementary material for: Virtual Reality in Clinical Nursing Practice Over the Past 10 Years: Umbrella Review of Meta-Analyses
Source: JMIR Serious Games. 2023 Nov 23;11:e52022. doi: 10.2196/52022 (PMC10690102; doi:10.2196/52022)
Supplement: Multimedia Appendix 2 [file games-v11-e52022-s004.docx]

**Table S1.** Distribution of VR application in clinical nursing.

| **Population** | **Frequency,proportion(n/%)** |
| --- | --- |
| neuronursing | 25(33.78%) |
| Pediatrics | 13(17.58%) |
| Surgical and Wound care | 11(14.86%) |
| oncological | 11(14.86%) |
| gerontic | 10(13.51%) |
| Others | 4(5.41%) |

**Table S2.** Distribution of outcome indicators of VR application in clinical nursing.

| **Outcomes** | **Frequency,proportion(n/%)** |
| --- | --- |
| pain | 37(17.29%) |
| anxiety | 36(16.82%) |
| cognitive function | 17(7.94%) |
| balance | 16(7.48%) |
| depression | 16(7.48%) |
| motion | 12(5.61%) |
| participation of life | 12(5.61%) |
| Mobility | 11(5.14%) |
| quality of life | 11(5.14%) |
| Satisfaction | 8(3.74%) |
| physiological& biochemical indicators | 8(3.74%) |
| Fatigue/tiredness | 8(3.74%) |
| fear | 7(3.26%) |
| others | 15(7.01%) |
